# Selective Serotonin Reuptake Inhibitors for Cessation of Betel Quid Use in Patients with Major Depressive Disorder in Taiwan

**DOI:** 10.3390/biomedicines12112633

**Published:** 2024-11-18

**Authors:** Chung-Chieh Hung, Hung-Pin Tu, Chia-Min Chung

**Affiliations:** 1 School of Medicine, Chung Shan Medical University, Taichung 40201, Taiwan; cshy2135@csh.org.tw; 2 Department of Psychiatry, Chung Shan Medical University Hospital, Taichung 40201, Taiwan; 3 Department of Public Health and Environmental Medicine, School of Medicine, College of Medicine, Kaohsiung Medical University, Kaohsiung 80708, Taiwan; 4 Department of Medical Research, Kaohsiung Medical University Hospital, Kaohsiung 80708, Taiwan; 5 Graduate Institute of Biomedical Sciences, China Medical University, Taichung 40402, Taiwan; 6 Department of Psychiatry and Center for Addiction and Mental Health, China Medical University Hospital, Taichung 40447, Taiwan

**Keywords:** abstinence therapy, antidepressant, betel quid use disorder, major depressive disorder

## Abstract

**Background/Objectives**: Major depressive disorder (MDD) frequently co-occurs with substance use disorders such as alcohol and nicotine use disorders. Comorbid substance use disorders worsen the clinical symptoms of MDD and exacerbate addictive behaviors and presentations. However, the relationship between MDD and betel quid use disorder (BUD) in Taiwan has not been extensively investigated. **Methods**: We performed this cross-sectional study investigated associations between betel quid use, BUD, and MDD specifically in the Taiwanese population. Long-term betel quid use is a major public health concern, contributing significantly to the high incidence of oral cancers, which rank fifth among the top ten most common cancers in Taiwan. **Results**: Among patients with MDD, the current BUD prevalence rate was 7.32%, and the lifetime BUD prevalence rate was 15.45%. Patients with comorbid BUD were more likely to have severe alcohol and nicotine dependence disorders and required longer antidepressant treatment. **Conclusions**: Notably, 16.98% of patients with comorbid BUD who received selective serotonin reuptake inhibitor treatment achieved abstinence. BUD has a detrimental effect on health outcomes in patients with MDD, and selective serotonin reuptake inhibitor treatment may be required to be prolonged for betel quid abstinence therapy to be effective. Additional studies should investigate medication therapies for betel quid addiction disorders.

## 1. Introduction

Major depressive disorder (MDD) is a serious psychiatric condition with a substantial global health care burden [1]. Among patients with MDD, substance use disorder is one of the most prevalent comorbidities [2]. The prevalence of substance use disorder in patients with MDD ranges from 12% to 80% [3,4,5]. Such comorbidity worsens clinical outcomes and increases suicide risk [6].

Betel quid (BQ) use disorder (BUD) is prevalent in many Asian countries [7]. BUD is diagnosed using criteria from the *Diagnostic and Statistical Manual of Mental Disorders, Fifth Edition* (*DSM-5*) [8]. The prevalence of BUD in patients with MDD is unknown, and the relationship between BUD, BQ use, and clinical outcomes in patients with MDD has not been well studied.

Notably, the addictive properties of BQ have not been well studied [7,9,10], and the exact mechanism of BQ addiction is unknown. One study highlighted the role of monoamine genotypes in heavy BQ use [11]. Another study suggested that monoamines play a key role in the addictive properties of BQ [12].

Alcohol and nicotine abuse have been demonstrated to worsen the clinical course of MDD [13,14,15,16,17]. As discovered, the available literature in this domain was restricted, illustrating an intricate and diverse connection. Although smoking might provide temporary relief for certain symptoms, it can also play a role in the emergence or exacerbation of adverse symptoms in the long term [18].

Acute alcohol intake reduces glutamate in the brain, and a subsequent rebound in glutamate release can disrupt monoamine pathways, exacerbating anxiety and depression [19]. Similarly, smoking can worsen MDD symptoms [20], and patients with comorbidities tend to increase their smoking habits [21].

No study has explored the effects of BQ on MDD severity or the potential for abstinence therapy in the treatment of BUD among patients with MDD. However, BQ dependence has been reported among Indo-Asian immigrants in the United Kingdom [22,23].

BQ is the fourth most popular psychoactive substance in the world [24]; therefore, its influence on patients with MDD warrants consideration. BQ is also classified as a Group I human carcinogen. BQ significantly increases the risk of oral potentially malignant disorders [25] and cancers in the oral cavity and pharynx [26,27]. Oral cancer associated with betel quid (BQ) use has been identified as a significant public health issue in Taiwan [28]. It ranks fifth among the most common cancers in Taiwan [29,30], and reducing harm from persistent BQ use remains a significant challenge for the Taiwanese government. Understanding the relationship between betel quid use disorder (BUD) and major depressive disorder (MDD) is essential for developing effective interventions to assist patients in abstaining from betel quid use and to improve overall health outcomes in individuals with MDD in Taiwan.

An observational study in Taiwan suggested that antidepressants may reduce BQ consumption [31], and a clinical trial demonstrated that antidepressants could effectively reduce BQ use within 2–4 weeks [32]. Nevertheless, the relationships between BQ use, BUD, and MDD remain poorly understood. No study has comprehensively investigated the associations and interactions between BUD and MDD.

Notably, no effective, evidence-based medications for BUD have been developed. The relationships between BQ use, BUD, and MDD must be clarified to better understand their effects on patients and to develop effective interventions to achieve abstinence. BQ use has been associated with carcinogenic risk in the oral cavity and pharynx; therefore, achieving abstinence from BQ use and treatment of BUD and MDD together could improve clinical outcomes in patients with MDD.

We conducted a cross-sectional study to investigate different clinical presentations in patients with MDD with or without BQ use habits and examined the effects of antidepressant treatments on BQ use and various health outcomes.

## 2. Results and Discussion

### 2.1. Study Design and Participant Demographic Information

**Figure 1:**
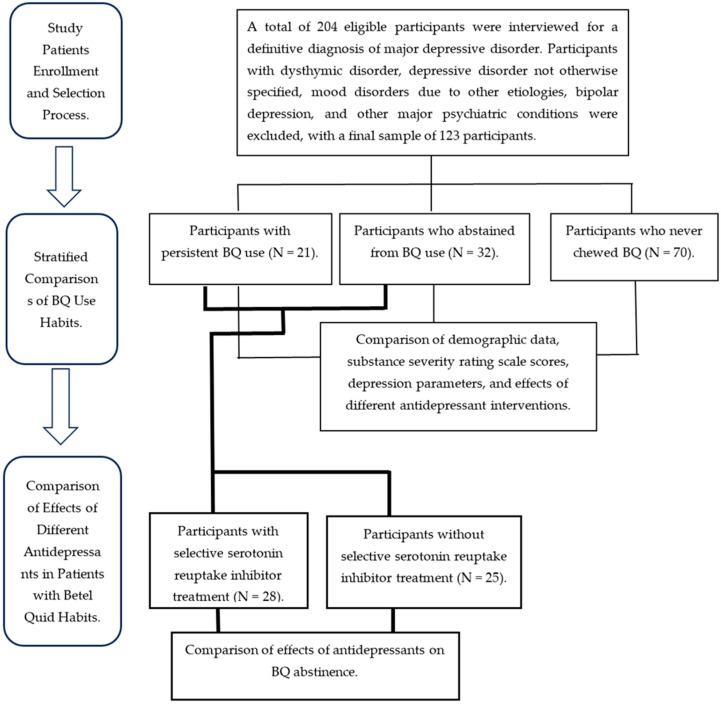
Patient selection process and overall study design.

The study design, including how patients were selected and grouped for statistical analyses, is depicted in Figure 1. A total of 204 participants were interviewed, and 81 participants were subsequently excluded because they did not meet the criteria for MDD. Data are presented as means (standard deviations) and are listed in Table 1. Among the participants with a BQ use habit, the average age was 47.34 (±10.5) years, the average length of education was 9.70 (±2.30) years, the average oral hygiene visual analog scale score was 2.37 (±0.66), the average substance use severity rating scale (SUSRS) for alcohol score was 1.04 (±2.65), the average SUSRS for tobacco score was 2.21 (±2.77), the average Hamilton depression rating scale (HAMDRS) score was 13.23 (±7.95), and the average antidepressant treatment duration was 5.94 (±5.86) years. Among the participants without a BQ use habit, the corresponding data points were 40.53 (±13), 10.11 (±2.39), 1.87 (±0.61), 0.24 (±0.84), 1.23 (±2.41), 9.94 (±6.73), and 1.95 (±2.77). The differences in age, oral hygiene visual analog scale score, SUSRS-alcohol score, SUSRS-tobacco score, HAMDRS score, and antidepressant treatment duration between the two groups were all significant.

### 2.2. Comparisons of Clinical Characteristics Among Participants Without a BQ Habit, with Persistent BQ Use, and with BQ Abstinence

The ages of the participants without a BQ habit, with persistent BQ use, and with BQ abstinence were 40.53 (±169.04), 42.76 (±100.29), and 50.34 (±96.81) years, respectively. Data on SUSRS-alcohol, SUSRS-tobacco, and HAMDRS scores and antidepressant treatment duration in the groups are presented in Table 2. The differences in the age and antidepressant treatment duration between the groups were significant.

### 2.3. Effects of Antidepressants on BQ Abstinence

We performed a summation of participants with persistent BQ use and those abstained from BQ use. The total 53 participants were divided into two groups according to treatment with SSRI or without SSRI. The effects of antidepressant treatment type on BQ abstinence are presented in Table 3. Antidepressant treatments were classified as selective serotonin reuptake inhibitors (SSRIs) or non-SSRIs. The SSRI group had an average age of 49.68 (±10.46) years, an oral hygiene score of 2.39 (±0.67), an SUSRS-alcohol score of 0.50 (±1.18), an SUSRS-tobacco score of 1.79 (±2.35), an SUSRS-BQ score of 1.14 (±2.79), a BQ use duration of 22.36 (±11.93) years, an HAMDRS score of 11.46 (±6.57), and an antidepressant treatment duration of 7.96 (±5.96) years. The non-SSRI group had an average age of 44.72 (±9.69) years, an oral hygiene score of 2.32 (±0.55), an SUSRS-alcohol score of 1.60 (±3.53), an SUSRS-tobacco score of 2.68 (±3.06), an SUSRS-BQ score of 1.28 (±2.62), a BQ use duration of 19.88 (±9.58) years, an HAMDRS score of 15.20 (±8.70), and an antidepressant treatment duration of 3.75 (±4.65) years.

**Figure 2:**
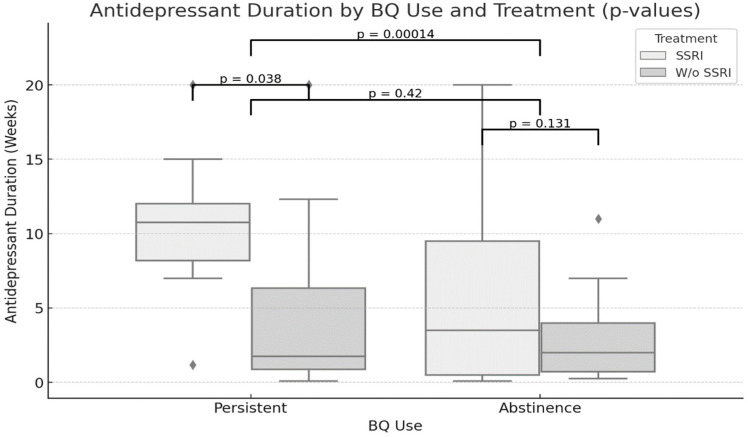
Comparison of antidepressant duration across betel quid use patterns and antidepressant treatment types in participants with major depressive disorder. Abbreviations: BQ, betel quid; SSRI, selective serotonin reuptake inhibitor; W/o, without. *p*-values represent comparisons between groups. *p* < 0.05 is considered significant.

Statistical significance was found across different betel quid use patterns and types of antidepressant treatment. The difference in antidepressant treatment duration between the groups was significant (Figure 2). Furthermore, among the participants with persistent BQ use, SSRI use was significantly associated with treatment duration.

### 2.4. Effects of Antidepressant Treatment Type on BQ Abstinence

We examined the effects of the antidepressant treatment type on BQ abstinence in 53 participants with comorbid BUD and BQ use habits (Table 4 and Table 5). Among the participants with comorbid BUD, the mean age was 41.11 (9.40) years, the SUSRS-alcohol score was 1.89 (3.78), the SUSRS-tobacco score was 3.33 (2.36), the HAMDRS score was 14.78 (4.73), and the mean antidepressant treatment duration was 8.7 (6.06) years. By comparison, among the participants without a BQ use habit, the mean age was 40.53 (12.91) years, the SUSRS-alcohol score was 0.24 (0.84), the SUSRS-tobacco score was 1.23 (2.39), the HAMDRS score was 9.94 (6.69), and the mean antidepressant treatment duration was 2.28 (3.21) years. Among the participants with a BQ use habit, the mean age was 48.61 (10.14) years, the SUSRS-alcohol score was 0.86 (2.82), the SUSRS-tobacco score was 1.98 (2.76), the HAMDRS score was 13.14 (8.34), and the mean treatment duration was 5.42 (5.55) years. Significant differences were observed between the groups in age, SUSRS-alcohol score, SUSRS-tobacco score, HAMDRS score, and antidepressant treatment duration. A χ^2^ test was performed to compare outcomes between participants receiving SSRIs and those not receiving SSRIs. The results revealed that participants receiving SSRIs had higher rates of BUD abstinence (16.98%) and BQ habit abstinence (28.30%) compared with those not receiving SSRIs. These findings suggest that SSRI treatment may be more effective than other antidepressant treatments in promoting BQ abstinence among patients with MDD.

### 2.5. Discussion

Our study investigated differences in the presentations and outcomes among individuals with MDD with or without BQ use habits. In total, 123 participants with MDD had a BQ use habit. The participants with a BQ use habit tended to be older, had poorer oral hygiene, had higher scores on the SUSRSs for alcohol and tobacco, had more severe depressive symptoms as measured using the HAMDRS, and required longer antidepressant treatment durations. Additionally, participants who did not stop BQ use had more severe depressive symptoms and longer antidepressant treatment durations compared with those without a BQ use habit. Regarding antidepressant treatment type, SSRIs and longer treatment durations were significantly associated with persistent BQ use.

The prevalence of BUD was 7.32% (9/123). The prevalence of lifetime BUD was 15.45% (19/123). Among the participants with BUD, with a BQ use habit, and without a BQ use habit, those with BUD had more severe alcohol and tobacco dependence and more depressive symptoms and required more time receiving antidepressant treatment. Notably, 16.98% of participants with BQ abstinence had BUD and were receiving SSRI treatment.

BQ induces feelings of well-being and euphoria [33]. The main ingredient of BQ is arecoline. Arecoline—one of the most crucial alkalines—influences carcinogenesis in the oral cavity [34] and cellular monoamine neurotransmission [9,11]. A study investigated the association between monoamine oxidase A gene polymorphism rs5953210 and BUD [12]. However, the mechanism by which the monoamine oxidase A gene influences substance use disorders is not fully understood. Notably, genetic factors may be associated with BQ addiction.

Monoamine oxidase A has been implicated in the pathogenesis of MDD. Nevertheless, the role of this enzyme in the delayed or refractory response to antidepressants is a topic of debate [35]. Additionally, substance abuse or dependence co-occurring with MDD has been associated with increased severity and recurrence of both conditions [36]. Several studies have investigated the negative outcomes of comorbid alcohol abuse or dependency [13,37,38]; however, no study has specifically addressed the effects or outcomes of BQ use and BUD in patients with MDD.

A study demonstrated that citalopram—a common SSRI—was associated with a remission rate of 28% to 33% and a response rate of 47% in patients with chronic and recurrent MDD [39], and clinical evidence supports the use of SSRIs as a first-line treatment for MDD [40,41]. The main goals in managing MDD are achieving remission and restoring patients’ function and quality of life [42]. SSRIs, which increase serotonin levels, have been effective in patients with alcohol dependence [43,44,45]. In addition, serotonin receptors play a role in the treatment of nicotine dependence [46]. Arecoline—the main component of BQ—increases serotonin levels by preventing neurotransmitter breakdown and increasing dopamine and serotonin concentrations inside neurons [47]. A study revealed that antidepressants can help with BQ abstinence [31], and a clinical trial demonstrated that SSRIs could achieve a 6-week or longer cessation rate of 34.2% in participants with a pure diagnosis of BUD [32].

The mechanism underlying BQ addiction is not known, and no evidence-based drugs have been developed for treating patients with BUD. Although SSRIs were reported to be effective in BQ cessation therapy [32], no study has examined their effects on BQ use in patients with MDD. Our study revealed that patients with MDD and BUD had worse outcomes and more severe clinical features. The interaction between BQ addiction and MDD is complex because substance dependence often involves deep-seated and long-lasting memories [48,49,50]. Our findings indicate that patients with BUD and MDD can be effectively treated using SSRIs. However, SSRI treatment must be implemented for longer among patients with both BUD and MDD than among those with only MDD. Cultural and social acceptance of BQ use complicates abstinence efforts [51,52,53].

Our study is the first to reveal a higher lifetime and current prevalence of BUD in patients with MDD. We found that these patients were more depressed, were more likely to become addicted to alcohol or tobacco, and required longer SSRI treatment. SSRIs were demonstrated to be effective in contributing to abstinence therapy in patients with MDD and BUD.

Early detection of BQ use and potential BUD in patients with MDD can aid clinicians in improving health outcomes. The potential for dual diagnoses of BUD and MDD is critical, as they alert clinicians to the likelihood of poorer outcomes and underscore the need for BQ abstinence. In addition, a longer duration of SSRI treatment might help reduce the burden of dual comorbidity in patients with MDD. Because no objective tools have been developed for diagnosing BUD, future studies should focus on developing more effective therapeutic drugs and personalized medicine for BQ addiction.

### 2.6. Study Limitations

This study has several limitations. First, the cross-sectional design of this study introduced several constraints. We were not able to determine a cause-and-effect relationship between MDD and BUD. In addition, most of the data were collected using psychometric questionnaires and rating scales; objective tools and biomarkers must be identified for diagnosing BUD. This study also had a small sample size. Despite these limitations, as a pioneering investigation into the association between antidepressants (especially SSRIs), BUD, and MDD, this study provides valuable preliminary evidence. Larger studies are required to replicate and validate these findings.

## 3. Materials and Methods

### 3.1. Study Participants

This study recruited participants from the outpatient psychiatry department at China Medical University Hospital in Taichung, Taiwan, between January 2014 and December 2016. A total of 204 patients with a history of anxiety or depression were enrolled and interviewed. Of these individuals, 81 were excluded because they did not have MDD. In total, 123 participants were enrolled. Data on their basic demographic characteristics were collected, and psychiatric diagnoses and clinical features related to BQ use were assessed. All participants provided informed consent and underwent clinical interviews conducted by a psychiatrist. MDD and BUD were diagnosed on the basis of *DSM-5* criteria. For MDD to be diagnosed, 5 or more symptoms must be present every day during a single 2-week period, with at least 1 symptom being either a depressed mood or a loss of interest or pleasure. The symptoms that are considered are (1) depressed mood, (2) markedly diminished interest or pleasure, (3) significant weight loss when not dieting or weight gain or decrease or increase in appetite, (4) insomnia or hypersomnia, (5) psychomotor agitation or retardation, (6) fatigue or loss of energy, (7) feelings of worthlessness or excessive or inappropriate guilt, (8) diminished ability to think or concentrate or indecisiveness, and (9) recurrent thoughts of death, recurrent suicidal ideation without a specific plan, or a suicide attempt or a specific plan for committing suicide.

The diagnosis of BUD was determined by the presence of at least 2 of the following 11 symptoms within the past year: (1) extensive or prolonged BQ consumption, (2) unsuccessful attempts to reduce BQ use, (3) substantial time spent chewing, (4) BQ cravings, (5) neglect of major responsibilities, (6) social or interpersonal problems, (7) abandoning activities, (8) hazardous use, (9) continued use despite awareness of problems, (10) tolerance, and (11) withdrawal.

Participants were excluded if they (1) abused illegal substances (e.g., heroin and amphetamines), (2) had other major psychiatric disorders (e.g., schizophrenia, bipolar disorder, and antisocial personality disorder), (3) had organic brain conditions (e.g., cerebrovascular disease, brain tumor, and head injury), (4) had any form of cancer or cancer-related disease, or (5) were unable to understand or speak Chinese. The study psychiatrist conducted semi-structured diagnostic interviews and reviewed the literature on psychiatric and addictive disorders [54,55]. Diagnoses of cancerous diseases were made on the basis of clinical history taken by the study psychiatrist. In addition, neurological or other brain symptoms were suitably met and excluded from our participants. Information regarding the initial age of BQ consumption, daily BQ consumption amount, weekly consumption frequency, and age of abstinence was collected. Among the participants with a BQ use habit, diagnoses of BUD were made in accordance with *DSM-5* criteria. Oral hygiene was assessed by using a visual analog scale and rated subjectively from good to bad (0 to 2). This study was approved by the China Medical University and Hospital Research Ethics Committee (CMUH103-REC1-059).

### 3.2. Psychometric Measures of Addiction Severity

Diagnoses of BUD were made in accordance with validated *DSM-5* criteria [7]. The SUSRS was used to evaluate BQ and alcohol consumption and smoking habits. The SUSRS was developed using *DSM-IV* and *International Classification of Diseases, Eleventh Revision* criteria, which comprises 21 items that measure the severity of substance use addiction [52]. SUSRS questionnaires have been widely used to evaluate alcohol consumption, smoking habits, and use of other drugs [56,57]. In this study, items assessing substance use were rated using yes-or-no questions, with each receiving a score of 1 (yes) or 0 (no). Clinical symptoms and signs of depression were rated using the HAMDRS [58].

### 3.3. Subgroups Analyses

The participants were divided into three groups on the basis of BQ use. Among the participants, 21 had persistent BQ use, 32 had abstained from BQ use, and 70 had never used BQ. Between-group differences were analyzed using a *t* test. Among the 21 participants with persistent BQ use, 9 met the criteria for BUD. The current prevalence of BUD in our study was 7.32%, and the lifetime prevalence of BUD was 15.45%.

We further stratified participants with a BQ habit into persistent and abstinent BUD cases. We then analyzed the type of antidepressant used and the overall duration of treatment among the 53 cases, categorizing them into 3 groups: non-abstinent BUD, abstinent BUD, and BQ habit abstinence.

### 3.4. Methods of Statistical Analysis

Statistical analyses were performed using SAS version 9.4 software (Cary, NC, USA). A *t* test was applied to compare the demographic information, BQ use conditions, and the effects of antidepressant treatment in patients with and without BQ use. We further analyzed the effects of antidepressant treatment type by dividing participants into 2 groups on the basis of whether they received SSRI treatment. A *t* test was then conducted to evaluate the association between treatment duration and antidepressant treatment type.

## 4. Conclusions

The findings of this study highlight the high prevalence of comorbid betel quid use disorder (BUD) in patients with major depressive disorder (MDD) in Taiwan. Betel quid addiction exacerbates depressive symptoms and diminishes the beneficial effects of antidepressant treatment in patients with MDD in Taiwan, underscoring the significant challenge of addressing BUD as a comorbidity in this population. Selective serotonin reuptake inhibitors (SSRIs) may play a role in abstinence therapy for patients with BUD. Longer-term interventions are required to fully investigate these effects.

## Figures and Tables

**Table 1:** Demographic and clinical characteristics of participants stratified by BQ use.

Variables	Participants with BQ Chewing Habits (*N* = 53)	Participants Without BQ Chewing Habits (*N* = 70)	*p*-Value
Age (years old)	47.34 (10.5)	40.53 (13)	0.01 *
Education (years)	9.70 (2.30)	10.11 (2.39)	0.17
Oral Hygiene	2.37 (0.66)	1.87 (0.61)	<0.001 **
SUSRS-alcohol	1.04 (2.65)	0.24 (0.84)	<0.001 **
SUSRS-tobacco	2.21 (2.77)	1.23 (2.41)	0.019 *
HAMDRS	13.23 (7.95)	9.94 (6.73)	0.007 *
Duration of Antidepressant Treatment (years)	5.94 (5.86)	1.95 (2.77)	<0.001 **

All data are illustrated as means (standard deviations). Abbreviations: BQ, betel quid; HAMDRS, Hamilton depression rating scale; SUSRS, substance use severity rating scale; *: *p* < 0.05; **: *p* < 0.001.

**Table 2:** Demographic and clinical characteristics of participants stratified by BQ use patterns.

Variables	No BQ Use (*N* = 70)	Persistent BQ Use (*N* = 21)	*p*-Value	Abstinent BQ Use (*N* = 32)	*p*-Value ^+^
Age (years old)	40.53 (169.04)	42.76 (100.29)	0.470	50.34 (96.81)	<0.001 **
SUSRS-alcohol	0.24 (0.71)	1.29 (9.21)	0.011 *	0.88 (5.79)	0.052
SUSRS-tobacco	1.23 (5.80)	2.57 (5.36)	0.026 *	1.97 (9.26)	0.189
HAMDRS	9.94 (45.39)	13.81 (50.96)	0.025 *	12.84 (72.72)	0.067
Duration of Antidepressant Treatment (years)	2.28 (10.48)	8.25 (37.41)	<0.001 **	6.70 (60.26)	<0.001 **

All data are illustrated as means (standard deviations). Abbreviations: BQ, betel quid; HAMDRS, Hamilton depression rating scale; SUSRS, substance use severity rating scale; *: *p* < 0.05; **: *p* < 0.001, statistical significance between No BQ use habits and persistent BQ ^+^ *p* values ^+^ represent comparisons between participants without BQ use and with BQ abstinence.

**Table 3:** Effects of antidepressant treatment type.

Variables	Treatment with SSRI (*N* = 28)	Treatment without SSRI (*N* = 25)	*p*-Value
Age (years old)	49.68 (10.46)	44.72 (9.69)	0.086
Oral Hygiene	2.39 (0.67)	2.32 (0.55)	0.675
SUSRS-alcohol	0.50 (1.18)	1.60 (3.53)	0.134
SUSRS-tobacco	1.79 (2.35)	2.68 (3.06)	0.244
SUSRS-BQ	1.14 (2.79)	1.28 (2.62)	0.86
BQ Use Duration	22.36 (11.93)	19.88 (9.58)	0.421
HAMDRS	11.46 (6.57)	15.20 (8.70)	0.088
Duration of Antidepressant Treatment (years)	7.96 (5.96)	3.75 (4.65)	0.007 **

Abbreviations: BQ, betel quid; HAMDRS, Hamilton depression rating scale; SSRI, selective serotonin reuptake inhibitor; SUSRS, substance use severity rating scale; **: *p* < 0.001.

**Table 4:** Demographic and clinical characteristics of patients with BUD, BQ use, and without BQ use.

Variables	No BQ Use (*N* = 70)	BUD Case (*N* = 9)	*p*-Value	BQ Habits (*N* = 44)	*p*-Value ^+^
Age (years old)	40.53 (12.91)	41.11 (9.40)	0.897	48.61 (10.14)	<0.001 **
SUSRS-alcohol	0.24 (0.84)	1.89 (3.78)	0.003 **	0.86 (2.82)	0.043 *
SUSRS-tobacco	1.23 (2.39)	3.33 (2.36)	0.016 *	1.98 (2.76)	0.131
HAMDRS	9.94 (6.69)	14.78 (4.73)	0.041 *	13.14 (8.34)	0.028 *
Duration of Antidepressant Treatment (years)	2.28 (3.21)	8.7 (6.06)	<0.001 **	5.42 (5.55)	<0.001 **

Abbreviations: BQ, betel quid; BUD, betel quid use disorder; HAMDRS, Hamilton depression rating scale; SUSRS, substance use severity rating scale. ^+^ *p*-value ^+^ represents comparisons between No BQ use and BQ habits. *: *p* < 0.05; **: *p* < 0.001.

**Table 5:** Antidepressant treatment type among participants with and without BUD abstinence.

Treatment	BUD Non-Abstinence (N = 9)	BUD Abstinence (N = 10)	BQ Habits Abstinence (N = 34)	χ^2^	*p*-Value
SSRI	7.55%	16.98%	28.30%	6.834	0.033 *
W/o SSRI	9.43%	1.89%	35.85%		

All data are presented as *N*%. Abbreviations: BQ, betel quid; BUD, betel quid use disorder; SSRI, selective serotonin reuptake inhibitor; W/o: without. *p*-values represent comparisons between groups, with *p* < 0.05 considered significant. *: *p* < 0.05.

## Data Availability

Data are not available because of privacy or ethical restrictions.
